# Polyphenol Stilbenes from Fenugreek (*Trigonella foenum-graecum* L.) Seeds Improve Insulin Sensitivity and Mitochondrial Function in 3T3-L1 Adipocytes

**DOI:** 10.1155/2018/7634362

**Published:** 2018-06-05

**Authors:** Gang Li, Guangxiang Luan, Yanfeng He, Fangfang Tie, Zhenhua Wang, Yourui Suo, Chengjun Ma, Honglun Wang

**Affiliations:** ^1^Center for Mitochondria and Healthy Aging, College of Life Sciences, Yantai University, Yantai 264005, China; ^2^Key Laboratory of Tibetan Medicine Research, Northwest Institute of Plateau Biology, Chinese Academy of Sciences, Xining 810008, China; ^3^College of Pharmacy, Qinghai Nationalities University, Xining 810008, China; ^4^University of Chinese Academy of Sciences, Beijing 100049, China

## Abstract

Fenugreek (*Trigonella foenum-graecum* L.) is a well-known annual plant that is widely distributed worldwide and has possessed obvious hypoglycemic and hypercholesterolemia characteristics. In our previous study, three polyphenol stilbenes were separated from fenugreek seeds. Here, we investigated the effect of polyphenol stilbenes on adipogenesis and insulin resistance in 3T3-L1 adipocytes. Oil Red O staining and triglyceride assays showed that polyphenol stilbenes differently reduced lipid accumulation by suppressing the expression of adipocyte-specific proteins. In addition, polyphenol stilbenes improved the uptake of 2-(N-(7-nitrobenz-2-oxa-1,3-diazol-4-yl)amino)-2-deoxyglucose (2-NBDG) by promoting the phosphorylation of protein kinase B (AKT) and AMP-activated protein kinase (AMPK). In present studies, it was found that polyphenol stilbenes had the ability to scavenge reactive oxygen species (ROS). Results from adenosine triphosphate (ATP) production and mitochondrial membrane potentials suggested that mitochondria play a critical role in insulin resistance and related signaling activation, such as AKT and AMPK. Rhaponticin, one of the stilbenes from fenugreek, had the strongest activity among the three compounds *in vitro.* Future studies will focus on mitochondrial biogenesis and function.

## 1. Introduction

**Figure 1:**
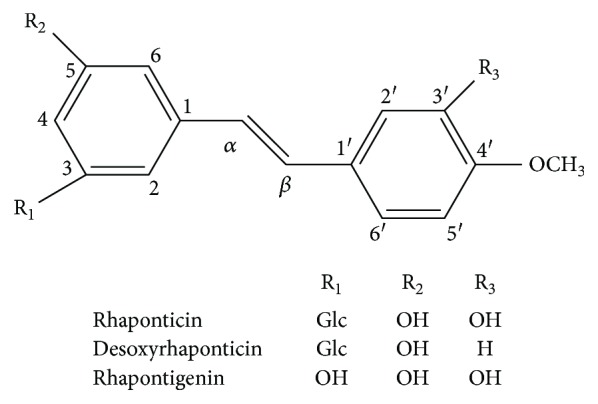
Chemical structure of polyphenol stilbenes from fenugreek.

Fenugreek (*Trigonella foenum-graecum* L.) is a well-known annual plant that belongs to the legume family and is widely distributed in China, India, and North African countries [1]. Fenugreek has been widely used as an edible vegetable and a medicinal plant for decades [2]. The seeds and some of its fractions have been reported to possess a wide range of biological and pharmacological effects [3, 4], including antioxidant [5, 6], hypoglycemic [7–10], hypercholesterolemia [11–13], and immunomodulatory activities [14]. Different beneficial functions of fenugreek are related to the variety of its natural components [15]. Fenugreek has been reported to contain galactomannans, nicotinic acid, alkaloids, flavonoids, salicylate, and amino acids [16]. Although several studies have shown that fenugreek seeds lowered blood glucose levels and improved lipid metabolism, the effective components are yet unknown. In our previous study, we successfully separated and purified several compounds from fenugreek seed extracts, including unsaturated fatty acids [17], flavonoids, and polyphenolic substances [18, 19]. To the best of our knowledge, it is the first report that polyphenol stilbenes (rhaponticin, desoxyrhaponticin, rhapontigenin) from *Trigonella foenum-graecum* L. seeds can be separated by high-speed counter-current chromatography (HSCCC) (Figure 1) [20].

Polyphenol compounds are composed of a group of substances with different chemical structures and activities and are widely present in nature [21]. Among a large variety of plant phenols, stilbenes have recently attracted extensive scientific attention. Resveratrol (3,5,4′-trihydroxy-trans-stilbene) is a well-known polyphenol compound that has widespread activities including antiobesity, antidiabetic, cardiovascular protective, and neuroprotective properties [22]. Recent studies have suggested that other polyphenol stilbenes may have a similar or even higher bioavailability compared to resveratrol [23]. Although several reports are available on the active components in fenugreek, little is known about the impact of polyphenol stilbenes on the glucose and lipid metabolism and its mechanisms of action have not yet been elucidated.

In our previous study, we demonstrated hypoglycemic effects of fenugreek extracts on streptozotocin- (STZ-) induced type 2 diabetic mice that were given with a high-fat diet. The results implied that the antidiabetic effects of stilbene extracts were related to their antioxidant effects [24]. Therefore, in the current study, we investigated the effects of polyphenol stilbenes from fenugreek seeds on both lipid accumulation and insulin resistance in 3T3-L1 adipocytes *in vitro*. To explore the underlying mechanisms of action, modulation of these stilbenes on the AMPK pathway and reactive oxygen species (ROS) was also discussed.

## 2. Materials and Methods

### 2.1. Differentiation of 3T3-L1 Preadipocytes and Induction of Insulin-Resistant Adipocytes

**Figure 2:**
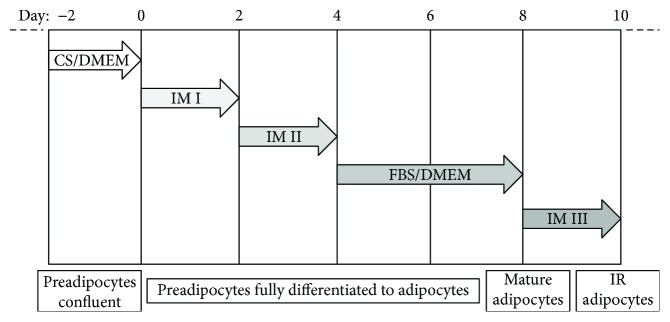
Timeline of 3T3-L1 differentiation and induction of insulin resistance. CS/DMEM: 10% calf serum/Dulbecco's Modified Eagle's Medium; IMI (induction media I): 10% fetal bovine serum (FBS)/DMEM + 0.5 mmol/L isobutylmethylxanthine (IBMX) + 1 *μ*mol/L dexamethasone (Dex) + 10 *μ*g/mL insulin; IMII (induction media II): 10% FBS/DMEM + 1 *μ*g/mL insulin; FBS/DMEM: 10% FBS/DMEM; IMIII (induction media III): 10% FBS/DMEM + 1 *μ*mol/L Dex.

3T3-L1 cells were obtained from the cell bank of the Institute of Biochemistry and Cell Biology of Shanghai (Shanghai, China). Preadipocytes were cultured in Dulbecco's Modified Eagle's Medium (DMEM) containing 10% calf serum (CS), 1.5 g/L sodium bicarbonate, and 1% penicillin-streptomycin solution. Cells were cultured at 37°C in a humidified atmosphere containing 5% CO_2_, and medium was replaced every other day until cells were confluent. As previously described [25], 3T3-L1 cells fully differentiated to mature adipocytes and induced to insulin-resistant adipocytes [26] (Figure 2).

### 2.2. Cell Viability Assay

All tested compounds were obtained from the Key Laboratory of Tibetan Medicine Research, Northwest Institute of Plateau Biology, Chinese Academy of Sciences (Xining, China) and included rhaponticin (RHAc), desoxyrhaponticin (dRHAc), and rhapontigenin (RHAg). Cell viability was assessed by the lactate dehydrogenase (LDH) assay. 3T3-L1 preadipocytes were seeded in 96-well plates (0.5 × 10^4^ cells/well) in DMEM medium without sodium pyruvate and containing 10% fetal bovine serum (FBS). Cells were incubated for 24 hr at 37°C until confluency and were separately treated with 0–100 *μ*mol/L RHAc, dRHAc, and RHAg for 48 hr. Then, medium was transferred to 1.5 mL microcentrifuge tubes and centrifuged at 12,000 ×g and 4°C for 10 min to remove any cell debris. A total of 500 *μ*L supernatant was added to the substrate solution, and the absorbance at 490 nm was measured using a spectrophotometer (Spectra MRTM, Dynex Technologies, Chantilly, VA, USA) according to the manufacturer's instructions of the LDH cytotoxicity assay kits (Beyotime Biotech Co., Beijing, China). The extracellular LDH activity in the media of normal control group was expressed as 100%.

### 2.3. Oil Red O Staining

To evaluate the effects of RHAc, dRHAc, and RHAg on lipid accumulation in 3T3-L1 adipocytes, cells were pretreated with 10 *μ*mol/L per compound for 2 days before maturation was initiated. 3T3-L1 preadipocytes were differentiated as mentioned above on Lab-Tek® chambered cover glasses (Nalge Nunc International, Naperville, IL, USA). After induction of insulin resistance and pretreatment of the compounds, respectively, 3T3-L1 mature adipocytes were gently washed with PBS and fixed in 10% neutral formalin. Cells were permeated using 0.5% Triton-X 100 and stained with filtered Oil Red O solution (60% isopropanol and 40% water) for 30 min at room temperature. Excess Oil Red O dye was removed by washing three times with 70% EtOH. Stained oil droplets in 3T3-L1 cells were imaged using a light microscope (Olympus, Japan).

### 2.4. Triglyceride Assay

To evaluate the intracellular triglyceride (TG) content, 3T3-L1 preadipocytes were cultured in 12-well plates as described under Section 2.3. At confluency, cells were washed twice in ice-cold phosphate-buffered saline (PBS) and harvested in ice-cold lysis buffer. Total TG content in the lysates was measured by using TG assay kits, and cellular protein was determined using the bicinchoninic acid (BCA) protein assay kits (Nanjing Jiancheng Bioengineering Institute, Nanjing, China).

### 2.5. Western Blot Analysis

As described under Section 2.3, 3T3-L1 preadipocytes were gently seeded into 6-well plates (1.5 × 10^5^ cells/well). Before maturation or inducing insulin resistance of 3T3-L1 adipocytes, cells were separately treated with RHAc, dRHAc, and RHAg for 2 days. Then, cells were washed trice with ice-cold PBS, harvested in 200 *μ*L lysis buffer, and lysates were centrifuged at 12,000 ×g for 20 min at 4°C. The protein content of the supernatant was determined using the BCA assay kit. Cell lysates were separated by sodium dodecyl sulfate-polyacrylamide (SDS-PAGE) gel electrophoresis, and proteins were transferred onto a polyvinylidene fluoride (PVDF) membrane using a Bio-Rad electrophoresis equipment. Membranes were blocked with 5% skim milk in Tris-buffered saline containing 0.05% Tween-20 (TBST) for 1 hr at room temperature and incubated overnight with primary antibodies (1 : 1000, Santa Cruz Biotechnology, CA, USA) at 4°C. Membranes were washed and incubated with a horseradish peroxidase- (HRP-) conjugated secondary antibody (Boster Biotech Co. Ltd., Wuhan, China) for 2 hr at room temperature. Membranes were washed and immunoreactive proteins were visualized using an enhanced chemiluminescent (ECL) assay kit (Beyotime Biotech Co., Beijing, China) according to the manufacturer's instructions. Protein bands were analyzed using a 5200 Multi Luminescent image analyzer (Tanon Science & Technology Co. Ltd. Shanghai, China).

### 2.6. 2-NBDG Uptake

As described under Section 2.1, 3T3-L1 preadipocytes were gently seeded into 12-well plates (0.8 × 10^5^ cells/well) and pretreated with RHAc, dRHAc, and RHAg for 2 days prior to induction of insulin resistance. Next, adipocytes were washed twice with PBS and the medium was changed to glucose-free DMEM containing 100 nmol/L of insulin. After 1 hr, 100 *μ*mol/L 2-NBDG was added to the medium and cells were incubated for another 30 min. Then, cells were washed three times with PBS, trypsinized, and collected in the dark. The fluorescence was measured (excitation at 485/20 nm and emission at 540/20 nm) using a FACSAria™ flow cytometer (Becton Dickinson, San Jose, CA, USA). The data are presented as the mean fluorescent signals for 20,000 cells.

### 2.7. ROS Detection

As described in Section 2.6, 3T3-L1 preadipocytes were seeded into 6-well plates (1.5 × 10^5^ cells/well) and cultured at 37°C. For induction of insulin resistance, 3T3-L1 adipocytes were treated with RHAc, dRHAc, and RHAg for 2 days. Then, cells were washed with freshly made, prewarmed PBS and incubated with 10 *μ*mol/L of 2′,7′-dichlorofluorescein diacetate (DCFH-DA) dye for 30 min at 37°C. Then, cells were washed twice with PBS and harvested with 0.25% trypsin solution. Released intracellular ROS was detected by a FACSAria flow cytometer at wavelengths of 470/530 nm (ex/em). The data are presented as the mean fluorescent signal of 20,000 cells.

As for the detection of the mitochondrial ROS, DCFH-DA was replaced by 5 *μ*M of MitoSOX reagent (Thermo Fisher Scientific, Beijing, China) and detected at wavelengths of 510/580 nm (ex/em).

### 2.8. Measurement of ATP Levels

Cells were pretreated as described in Section 2.7. After induction of insulin resistance and treatment with RHAc, dRHAc, and RHAg, cells were washed twice with ice-cold PBS and harvested with 0.25% trypsin solution. The level of ATP was determined by a spectrophotometer (Spectra MRTM, Dynex Technologies, Chantilly, VA, USA) and an ATP bioluminescence assay kit (Beyotime Biotech Co., Beijing, China) according to the manufacturer's instructions.

### 2.9. Monitoring of Mitochondrial Membrane Potential

Cells were pretreated as described in Section 2.8, harvested with 0.25% trypsin solution, and washed twice with PBS. Then, cells were resuspended in warm DPBS containing 10 *μ*M of JC-1 (Sigma-Aldrich) and incubated at 37°C for 30 min. Next, cells were washed once with prewarmed PBS and centrifuged (1000 ×g, 5 min). Then, cells were gently resuspended and analyzed on a FACSAria flow cytometer with 488 nm excitation.

### 2.10. Statistical Analysis

Data are presented as the mean ± SD from three independent experiments. Statistical analysis was performed by one-way ANOVA or Student's *t*-test using statistical analysis software SPSS version 18.0 (SPSS, Chicago, IL, USA). *P* < 0.05 was considered statistically significant.

## 3. Results

### 3.1. Cell Viability Assay

**Figure 3:**
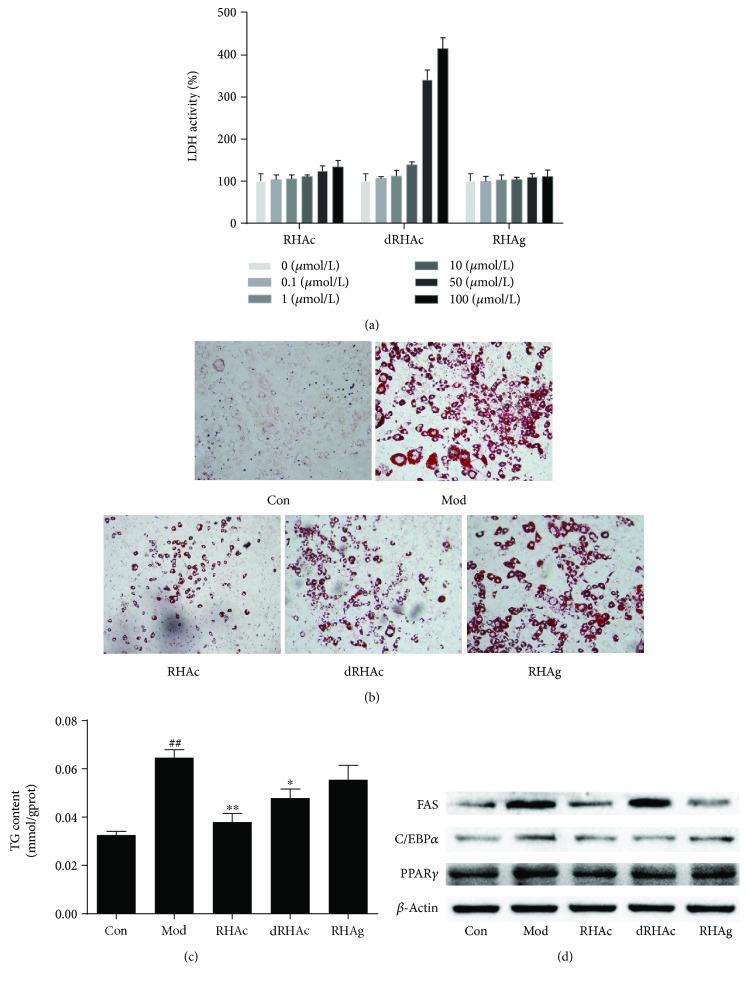
Effects of polyphenol stilbenes on viability and differentiation of 3T3-L1 adipocytes. (a) 3T3-L1 preadipocytes were incubated with different concentrations of polyphenol stilbenes for 48 hr after which cell viability was determined by extracellular LDH assay in the media. Data are expressed as the mean ± SD of three independent experiments. (b) 3T3-L1 preadipocytes were induced to full differentiation for 8 days as described in Figure 2. Lipid droplets were stained by Oil Red O. Con: undifferentiated 3T3-L1 preadipocytes; Mod: fully differentiated 3T3-L1 adipocytes. (c) On day 8, the cellular triglyceride (TG) content was measured and normalized against total protein. Data are presented as the mean ± SD of three independent experiments (*n* = 3). ^##^*P* < 0.01 mature adipocytes versus preadipocytes; ^∗^*P* < 0.05, ^∗∗^*P* < 0.01 compound-treated adipocytes versus mature adipocytes. (d) Adipocyte-specific protein expression on day 8 was determined by Western blot analysis.

The cytotoxicity of polyphenol stilbenes was measured by the extracellular LDH assay in the media (shown in Figure 3(a)). We found that polyphenol stilbenes did not have obvious effects on the viability of 3T3-L1 preadipocytes at the tested concentrations of 0.1–10 *μ*mol/L for 48 hr. Thus, the concentration of three polyphenol stilbenes were confirmed as 10 *μ*mol/L in the following experiments. And 1 *μ*mol/L rosiglitazone was used as a positive control.

### 3.2. Lipogenesis and TG Assay

During the differentiation of 3T3-L1 preadipocytes to adipocytes, the formation of lipid droplets is a typical phenomenon that is used as a marker of differentiation [27]. Lipid droplets in differentiated 3T3-L1 adipocytes were stained by Oil Red O.  Figure 3(b) shows that more big droplets were observed in fully differentiated 3T3-L1 cells when compared to undifferentiated control cells. This indicated that obvious lipogenesis occurred during differentiation. Treatment with both RHAc and dRHAc significantly decreased the accumulation of lipid droplets. To quantify the intracellular lipid content, TG levels were determined. The results presented in Figure 3(c) showed that during differentiation, treatment with RHAc, dRHAc, and RHAg significantly inhibited cellular TG accumulation (*P* < 0.05 or *P* < 0.01). The TG content in RHAc and dRHAc-treated cells was significantly lower compared with mature cells. These data indicated that polyphenol stilbenes differently reduced lipid accumulation during the differentiation of 3T3-L1 adipocytes.

### 3.3. Expression of Adipocyte-Specific Protein during 3T3-L1 Differentiation

Adipocyte differentiation from 3T3-L1 preadipocytes is associated with the expression of adipocyte-specific genes including FAS, C/EBP*α*, and PPAR*γ* [28]. Therefore, we investigated the expression of these proteins (Figure 3(d)). In preadipocytes, these proteins were all expressed at a low level and were significantly increased in mature adipocytes. In general, RHAc suppressed the expression of all these proteins, and dRHAc and RHAg increased the levels of FAS and PPAR*γ*. Together, these data indicated that the different effects of polyphenol stilbenes on the adipogenesis were tightly associated with their modulations on the expression of adipocyte-specific proteins during adipocyte differentiation.

### 3.4. 2-NBDG Uptake

**Figure 4:**
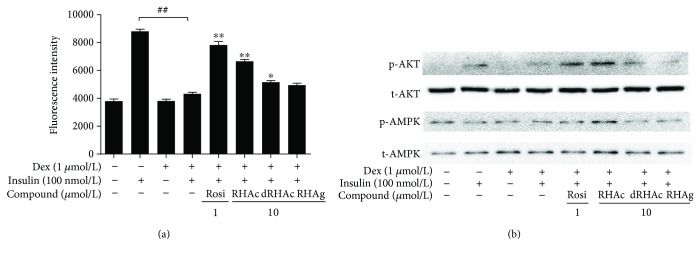
Effects of polyphenol stilbenes on 2-NBDG uptake and p-AKT and p-AMPK expression in 3T3-L1 adipocytes. (a) Results are presented as the means ± SD of three independent experiments (*n* = 3). ^##^*p* < 0.01 Insulin-resistant (IR) adipocytes versus mature adipocytes; ^∗^*P* < 0.05, ^∗∗^*P* < 0.01 compound-treated adipocytes versus IR adipocytes. (b) p-AKT and p-AMPK expression in 3T3-L1 adipocytes was determined by Western blot analysis.

To investigate the effects of polyphenol stilbenes on glucose uptake in insulin-resistant (IR) 3T3-L1 adipocytes, the fluorescent deoxyglucose analog (2-NBDG) was used to measure the rates of glucose uptake. 2-NBDG has been wildly used in various studies, especially for exploring cellular metabolic functions associated with GLUT systems [29]. As shown in Figure 4(a), 2-NBDG uptake in the IR group significantly decreased. Rosiglitazone (Rosi), known as a positive drug for the treatment of insulin resistance, significantly increased insulin-stimulated glucose uptake in 3T3-L1 adipocytes at the concentration of 1 *μ*mol/L. These data suggested that establishing the IR model in this study was successful. Treatment with RHAc and dRHAc enhanced the uptake of glucose compared with the IR group.

### 3.5. P-AKT and P-AMPK Expression

In previous studies, it has been reported that both Akt and AMPK signaling are critical for controlling metabolic disorders, especially in the insulin signaling cascade through glucose transport [30]. We determined the expression of total Akt and phospho-Akt Ser473 as well as total AMPK and phospho-AMPK Thr172 by Western blot analysis to elucidate how polyphenol stilbenes promote glucose uptake in IR 3T3-L1 adipocytes.

**Figure 5:**
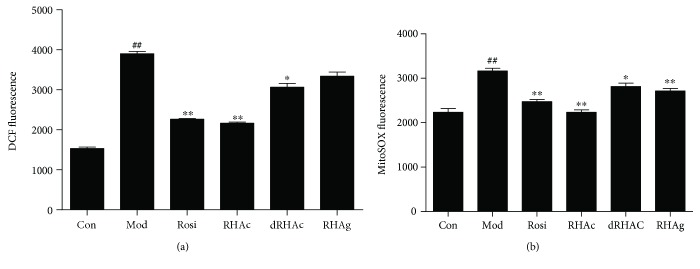
Effects of polyphenol stilbenes on ROS production in 3T3-L1 adipocytes. 1 *μ*mol/L of Rosi and 10 *μ*mol/L of the three polyphenol stilbenes were used in the experiments. Results are shown as the mean ± SD of three independent experiments (*n* = 3). ^##^*P* < 0.01, IR adipocytes versus mature adipocytes; ^∗^*P* < 0.05, ^∗∗^*P* < 0.01 compound-treated adipocytes versus IR adipocytes.

Data presented in Figure 4(b) demonstrate the effects of polyphenol stilbenes on the activation of AKT and AMPK. Our results demonstrated that both the phosphorylation of AKT and AMPK were significantly decreased in IR adipocytes. Insulin at 100 nmol/L significantly stimulated Akt and AMPK activity. Rosi (1 *μ*mol/L), a well-known AMPK activator used in this study significantly reversed the reduction of AMPK phosphorylation. Moreover, RHAc treatment showed a more remarkable effect than the other two compounds. As expected, we demonstrated that treatment with polyphenol stilbenes increased ATP levels in IR 3T3-L1 adipocytes (Figure 5(a)).

### 3.6. Detection of ROS Levels

Recent studies have suggested that several naturally derived active components could prevent metabolic diseases. During adipocyte proliferation and differentiation, the generation of ROS was related to the activation of AMPK [31]. Therefore, in this study, we evaluated the production of ROS by using DCFH-DA dye. As shown in Figure 5(a), intracellular ROS was rapidly upregulated in IR adipocytes. Under the same conditions, treatment with different compounds had different effects on the production of ROS. For example, Rosi and RHAc significantly reduced the expression of ROS (*P* < 0.01), whereas RHAg did not have obvious effects on intracellular ROS.

Mitochondria are the main providers but also the main scavengers of cell oxidative stress. Therefore, we monitored mitochondrial ROS by measuring the fluorescence of MitoSOX.  Figure 5(b) showed that mitochondrial ROS of IR adipocytes was significantly elevated by approximately 40% when compared with mature adipocytes. Treatment with Rosi and polyphenol stilbenes had obvious effects on the scavenging of mitochondrial ROS (*P* < 0.01 or *P* < 0.05). Taken together, RHAc had the most significant effect on inhibiting the generation of ROS, no matter it is intracellular or mitochondrial.

### 3.7. ATP Production and Mitochondrial Membrane Potential

**Figure 6:**
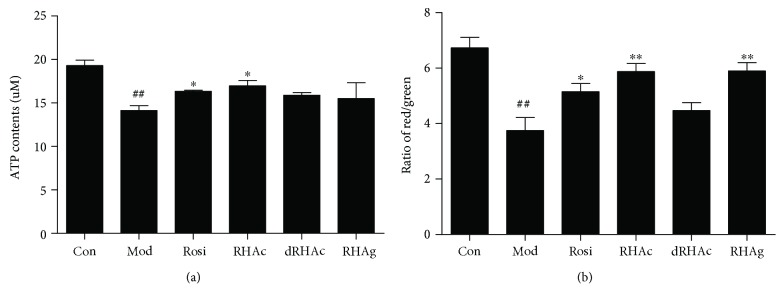
Effects of polyphenol stilbenes on ATP production (a) and mitochondrial membrane potential (b) in 3T3-L1 adipocytes. 1 *μ*mol/L of Rosi and 10 *μ*mol/L of the three polyphenol stilbenes were used in the experiments. Results are presented as the mean ± SD of three independent experiments (*n* = 3). ^##^*P* < 0.01, IR adipocytes versus mature adipocytes; ^∗^*P* < 0.05, ^∗∗^*P* < 0.01, compound-treated adipocytes *versus* IR adipocytes.

Mitochondria are the “power houses” of eukaryotic cells. The main function of mitochondria is to produce the cellular energy source, ATP. Thus, ATP production and the membrane potential can be used to evaluate mitochondrial function. The membrane-permeant JC-1 dye is widely used to monitor mitochondrial health. The JC-1 dye produces potential-dependent accumulation in mitochondria, as indicated by a shift in fluorescence emission from green (~529 nm) to red (~590 nm). Consequently, mitochondrial depolarization is indicated by a decrease in the ratio of red/green fluorescence intensity [32]. The data presented in Figure 6 suggested that the mitochondrial function of IR adipocytes was significantly damaged (*P* < 0.01). Both Rosi and RHAc could significantly increase ATP production and the mitochondrial membrane potential. However, the effects of RHAc were better compared to that of Rosi. These findings were consistent with the ROS scavenging ability of the compounds evaluated previously, which had been shown in Figure 5.

## 4. Discussion

Type 2 diabetes mellitus (T2DM) has become one of the world's most important public health problems. Insulin resistance seems to be attributed to the progressive failure of T2DM and metabolic syndrome [33]. Adipocytes play a central role in maintaining lipid homeostasis and energy balance [34]. Induced 3T3-L1 preadipocytes can differentiate into mature adipocytes, which are widely used to investigate glucose and lipid metabolism in adipocytes *in vitro* [35]. To fight against metabolic disorders, scientific research is focused on effective functional natural molecules from herbals, fruits, and vegetables [31]. Among these molecules, stilbenes, a group of polyphenols have received great interest in recent years. In our previous study, we demonstrated that extracts from stilbenes from fenugreek have beneficial hypoglycemic effects in diabetic mice, and three polyphenol stilbenes (RHAc, dRHAc, and RHAg) were separated from fenugreek seeds by HSCCC [20]. Therefore, in the current study, we further explored the effects of these polyphenol stilbenes on lipogenesis and glucose uptake in IR 3T3-L1 adipocytes.

The data presented here showed that these stilbenes could inhibit lipid accumulation during 3T3-L1 differentiation by suppressing adipocyte-specific proteins at a concentration of 10 *μ*mol/L. In addition, glucose uptake was differently improved after treatment with the three compounds. When comparing the effects of the compounds used, RHAc had the best effect. RHAc significantly stimulated the activation of Akt and AMPK in IR 3T3-L1 adipocytes. However, RHAg had the lowest activity despite its structure contains 3 phenolic hydroxyl groups.

It has been shown that the antiradical and the hydrogen peroxide scavenging activities by phenolic compounds positively correlate with both the position and number of hydroxyl groups bound with the aromatic ring [36, 37]. Moreover, structure-activity relationship analysis indicated that the therapeutic effects of natural phenols involve the reduction of ROS and include polyphenol stilbenes [38]. After the hypoglycemic activity of these stilbenes was confirmed, we further investigated both the intracellular and mitochondrial ROS production in IR 3T3-L1 adipocytes (Figure 5). Oxidative stress is closely linked with the development of T2DM [39] and ROS can suppress the insulin response and contribute to the development of insulin resistance, a key pathological feature of T2DM [40]. Our results regarding ROS measurements supported this conclusion. It seemed likely that the activation of AKT and AMPK was related to the production of ROS [30]. With regard to the polyphenol stilbenes, ROS generation in the RHAc-treated group was significantly reduced. This is consistent with the structure-activity relationship of these compounds in ROS scavenging. Moreover, compounds with a strong ROS clearing ability may also activate the AMPK and other related pathways [41]. Eventually, this would lead to inefficient modulation of the glucose and lipid metabolism, as demonstrated by RHAc in this study. Lipid accumulation and impaired glucose uptake are related to the weakened metabolic capacity of mitochondria in IR adipocytes. Several natural active products, such as RHAc improved mitochondrial function, enhanced insulin sensitivity, and promoted the metabolism of carbohydrates and fatty acids.

In conclusion, our results demonstrated that polyphenol stilbenes isolated from fenugreek seeds showed marked improvement in insulin sensitivity and mitochondrial function in 3T3-L1 adipocytes. RHAc possessed the best effects *in vitro*. Moreover, the results strongly indicated that mitochondria play a major role in insulin resistance and related signaling activation. In the future, we will focus on mitochondrial biogenesis and function to explore the underlying mechanism of action.
